# Clinical Manifestations and Diagnosis of Axial Spondyloarthritis

**DOI:** 10.1097/RHU.0000000000001575

**Published:** 2020-10-23

**Authors:** Jessica A. Walsh, Marina Magrey

**Affiliations:** From the ∗ University of Utah School of Medicine and Salt Lake City Veterans Affairs Medical Center, Salt Lake City, UT; † The MetroHealth System and School of Medicine, Case Western Reserve University, Cleveland, OH.

**Keywords:** ankylosing spondylitis, axial spondyloarthritis, diagnosis, inflammatory back pain, primary care physicians

## Abstract

**Background:**

Axial spondyloarthritis (axSpA) is a chronic, rheumatic disease characterized by inflammation of the sacroiliac joint, spine, and entheses. Axial spondyloarthritis affects up to 1.4% of adults in the United States and is associated with decreased quality of life, increased mortality, and substantial health care–related costs, imposing a high burden on patients, their caregivers, and society.

**Summary of Work:**

Diagnosing axSpA can be difficult. In this review, we seek to help rheumatologists in recognizing and diagnosing axSpA.

**Major Conclusions:**

A discussion of challenges associated with diagnosis is presented, including use and interpretation of imaging, reasons for diagnostic delays, differences in disease presentation by sex, and differential diagnoses of axSpA.

**Future Research Directions:**

The early diagnosis of axSpA and advances in available therapeutic options have improved patient care and disease management, but delays in diagnosis and treatment remain common. Additional research and education are critical for recognizing diverse axSpA presentations and optimizing management early in the course of disease.

Spondyloarthritis (SpA) encompasses a group of inflammatory diseases that may be referred to as ankylosing spondylitis (AS), reactive arthritis, psoriatic arthritis, juvenile SpA, SpA associated with inflammatory bowel disease, undifferentiated SpA, peripheral SpA, axial SpA (axSpA), nonradiographic axSpA (nr-axSpA), or radiographic axSpA.^1,2^ Spondyloarthritis with predominantly axial or peripheral involvement is termed axSpA (including nonradiographic and radiographic axSpA) or peripheral SpA, respectively.^1^ Axial SpA is a chronic disease that mainly involves the sacroiliac joints (SIJs) and spine.^3^ The National Health and Nutrition Examination Survey conducted in 2009–2010 estimated that the prevalence of axSpA ranges from 0.9% to 1.4% in the adult population in the United States.^4^ However, the true prevalence is unknown because of the significant delay in diagnosis, underrecognition of the disease, and challenges regarding case ascertainment in epidemiological data sets.^5^

Patients with axSpA commonly present with back pain that starts before 45 years of age.^6^ The characteristic features of the back pain include chronicity (>3 months), insidious onset, improvement with exercise, occurrence at night with improvement upon waking, and no improvement with rest (Fig. 1).^7–11^ Inflammatory back pain (IBP) criteria are important in screening for axSpA. The relatively high sensitivity of IBP (approximately 70%–95%) for axSpA among at-risk patients (back pain >3 months with onset age <45 years) renders it useful in axSpA screening.^11–13^ However, studies evaluating strategies for referral to a rheumatologist showed that only 17% to 33% of at-risk patients with IBP received an axSpA diagnosis, demonstrating that IBP criteria alone are not specific for diagnosis of axSpA.^11,12,14,15^

**FIGURE 1:**
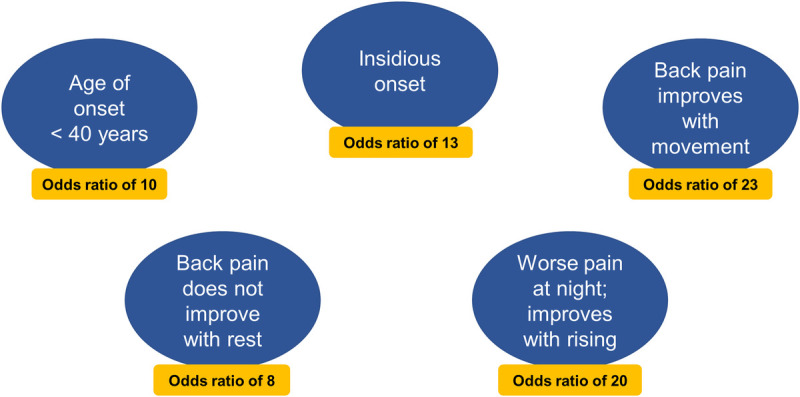
The 5 parameters that independently contribute to inflammatory back pain based on the ASAS criteria; inflammatory back pain requiring further investigation is usually indicated if 4 or more parameters are positive.^10^

The average symptom duration before diagnosis of axSpA has been reported to be as long as 13 years.^16^ The early diagnosis and subsequent treatment of axSpA may substantially decrease the burden of disease and increase quality of life; however, misdiagnoses, diagnostic delays, and underdiagnosis remain challenges.^5,17^ Additionally, sex differences have been described, with women experiencing longer delays in diagnosis,^18,19^ even though the age at disease onset is similar for men and women or even slightly earlier in women.^20,21^ In this narrative review, we seek to help rheumatologists in diagnosing axSpA. We also discuss current challenges with axSpA diagnosis, including use and interpretation of imaging, reasons for diagnostic delays, differences in disease presentation by sex, and differential diagnoses of axSpA.

## Concepts of axSpA and Common Disease Features

**FIGURE 2:**
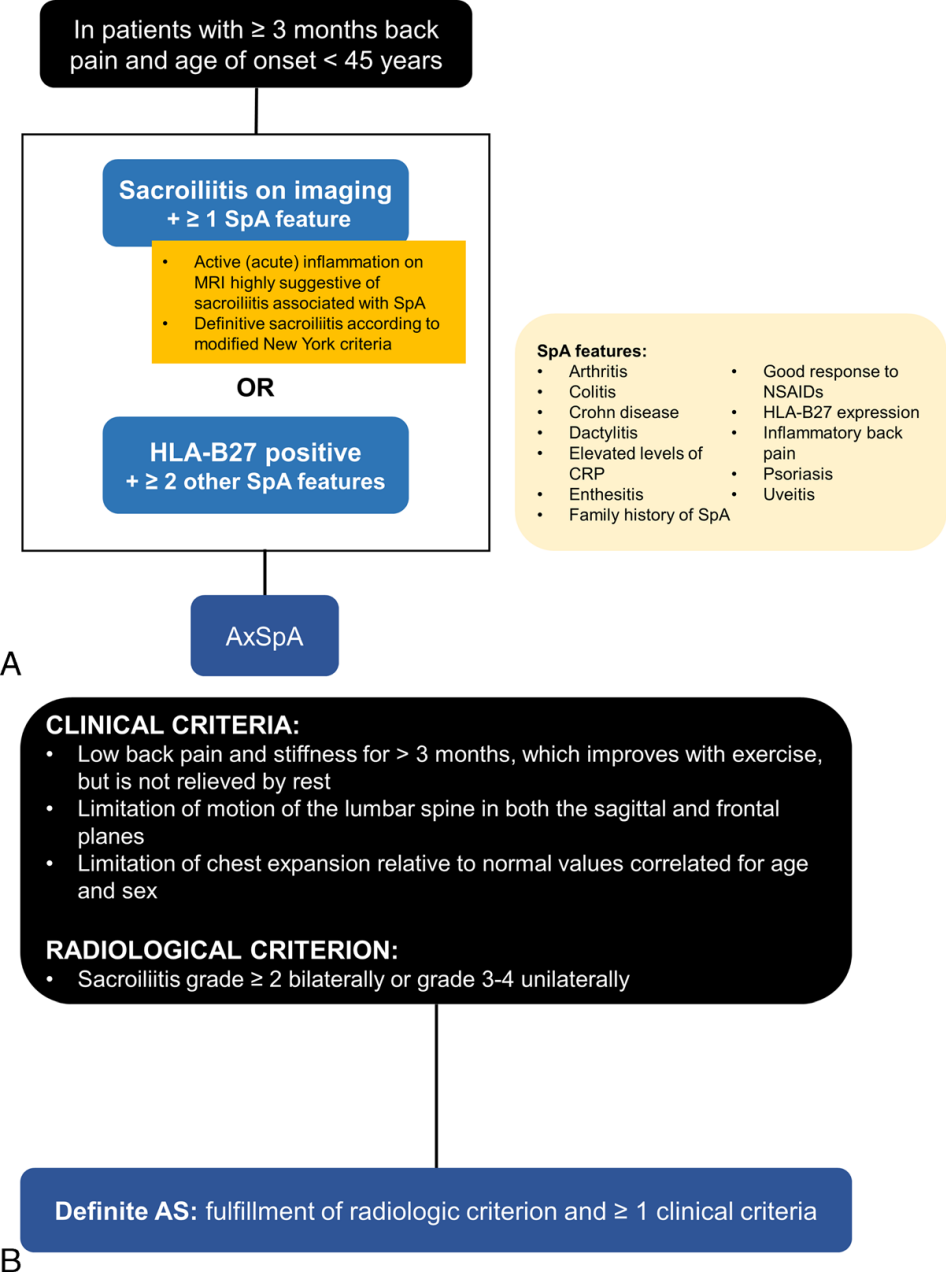
A summary of the (A) 2009 ASAS classification criteria for axSpA in patients presenting with chronic back pain lasting 3 months or more and age at onset of younger than 45 years^6,22^ and (B) 1984 modified New York criteria for AS. The ASAS criteria account for patients with and without radiographic sacroiliitis. Furthermore, patients meeting both the ASAS and modified New York criteria are classified as having AS.^6,24^

The Assessment of SpondyloArthritis international Society (ASAS) have developed classification criteria for axSpA,^6,22^ as well as peripheral SpA or SpA in general.^23^ The criteria for axSpA are based on the presence of sacroiliitis on imaging or human leukocyte antigen B27 (HLA-B27) positivity in the presence of other SpA features (Fig. 2A).^6,22^ Axial SpA with radiographic sacroiliitis is designated as radiographic axSpA, which overlaps with the definition of AS based on the 1984 modified New York criteria (Fig. 2B).^6,24^ Radiographic sacroiliitis is the hallmark of AS^25^; however, AS symptoms such as back pain, difficulty sleeping, and fatigue may be present for several years before radiographic changes are detected. Axial SpA without definitive radiographic sacroiliitis is termed nr-axSpA. Patients with nr-axSpA may or may not have sacroiliitis on magnetic resonance imaging (MRI).^26^

The conceptual paradigm of axSpA has been evolving. Nonradiographic axSpA and radiographic axSpA may be considered as 2 distinct but overlapping subtypes of axSpA. Axial SpA may also be conceptualized along a continuum of structural damage, with nonradiographic disease representing little or no structural damage and radiographic axSpA damage representing more advanced structural damage to the SIJs.^27,28^ Some patients initially classified with nr-axSpA will progress along the damage continuum to later fulfill criteria for radiographic axSpA^29,30^; studies have shown that 5% to 25% of patients with nr-axSpA progressed to radiographic axSpA within 2 to 8 years,^31–33^ and up to 28% progressed 10 years or more after diagnosis.^34^ Regardless, the overall burden of disease, as determined by disease activity, quality-of-life measures, and responses to treatment, is similar in patients with AS and those with nr-axSpA.^27,35–38^

In general, the diagnosis of axSpA is a clinical judgment based on features that, taken together, are characteristic of the disease.^39^ These features include diverse combinations of historical manifestations, physical findings, laboratory results, and imaging data. Common SpA features are listed in Table 1.^7–9^ Likelihood ratio estimates are also included for conceptualizing the relative importance of various SpA features for detecting axSpA (Table 1).^7–9^

**TABLE 1:** Likelihood Ratios (LRs) of SpA Features and Scoring Rubric to Guide the Diagnosis of AxSpA^7–9^

SpA Features	LR
Psoriasis	2.5
Increased levels of acute-phase reactants^a^	2.5
Inflammatory back pain	3.1
Heel pain (enthesitis)	3.4
Inflammatory bowel disease^b^	4.0
Peripheral arthritis	4.0
Dactylitis	4.5
Good response to NSAIDs	5.1
Family history of axSpA, reactive arthritis, inflammatory bowel disease, psoriasis, or uveitis	6.4
Iritis or anterior uveitis	7.3
HLA-B27 expression	9.0
Sacroiliitis by MRI	9.0

^a^Acute-phase reactants include erythrocyte sedimentation rate and CRP.

^b^Inflammatory bowel disease includes Crohn disease and ulcerative colitis.

Extra-articular manifestations such as uveitis, inflammatory bowel disease (including Crohn disease and ulcerative colitis), enthesitis, and psoriasis are relatively common in SpA diseases.^40^ Reports of the prevalence of uveitis among patients with axSpA range from 6% to greater than 30%.^28,41–45^ Uveitis in axSpA is an HLA-B27–associated disease characterized by episodes of acute, painful inflammation in the anterior chamber of the eye; it is an indicator of disease severity in axSpA and has been linked to worse physical function.^46^ The prevalence of inflammatory bowel disease is approximately 4% to 6% among patients with axSpA.^40^ Minimally symptomatic gut inflammation occurs in up to 60% of patients with AS, and up to 20% of these patients may develop Crohn disease within 5 years.^47–50^ Enthesitis refers to inflammation at the insertion sites of tendons, ligaments, or joint capsule fibers into bone,^51^ with a prevalence of approximately 35% to 60% in axSpA.^40,52,53^

Psoriasis affects approximately 10% of patients with axSpA.^28,40^ Some patients with psoriatic disease and axial involvement (axial psoriatic arthritis [axPsA]) may present differently than patients with other forms of axSpA. Age at onset may be older than 45 years in some patients, and IBP has been reported in approximately half of patients with axPsA.^54^ Sacroiliitis may be less symmetrical and of a lower grade with axPsA than AS, and syndesmophytes are more frequently paramarginal and bulky in axPsA.^55^ Furthermore, in AS, vertebral involvement typically begins in the lumbar spine or thoracolumbar junction and progresses caudally along contiguous vertebrae,^25^ whereas in axPsA, noncontiguous vertebrae may be affected, and isolated cervical involvement may occur.^55^ Importantly, up to one-third of patients with axPsA have spondylitis without sacroiliitis, whereas sacroiliitis is believed to occur before or in conjunction with spondylitis in patients with AS.^55,56^ Patients with axPsA have lower rates of HLA-B27 positivity, particularly patients with manifestations that differ from classic AS-like presentations.^55,56^ The relative impact of various axPsA subphenotypes is largely unknown, but the burden of axPsA (all subphenotypes) has been reported to be comparable to that of AS with respect to disease activity, function, and quality of life.^55–59^ Although additional research is required to better understand the spectrum of axPsA, improved awareness of the diversity of axPsA may influence recognition of axial involvement, particularly in patients who lack classic IBP or involvement of the SIJs.

## Diagnostic Considerations

**FIGURE 3:**
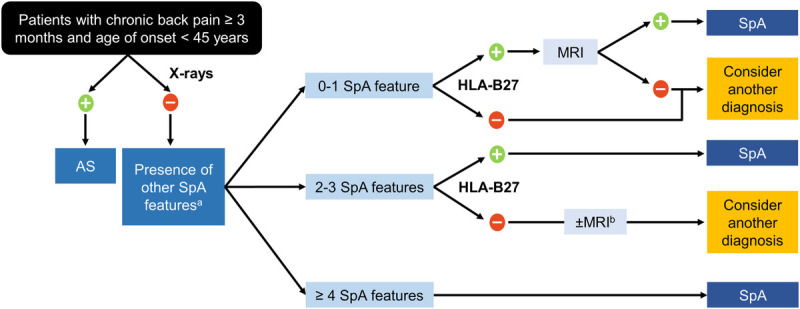
A diagnostic guide for axSpA among patients with chronic low back pain lasting 3 months or more and age at onset of younger than 45 years.^60^ A, Spondyloarthritis features include alternating buttock pain, dactylitis, asymmetrical arthritis, elevated acute-phase reactants (e.g., CRP or erythrocyte sedimentation rate), enthesitis, inflammatory back pain, inflammatory bowel disease, family history of SpA in a first- or second-degree relative, marked response to NSAIDs, psoriasis, and uveitis. B, The figure was modified from its original version to suggest that MRI of the sacroiliac joints may be appropriate in some patients who are x-ray– and HLA-B27–negative but who have 2 to 3 SpA features.

Diagnostic algorithms have been designed to guide rheumatologists in assessing clinical SpA features, imaging characteristics, and laboratory results. The algorithm shown in Figure 3 was tested in populations with chronic back pain (lasting >3 months) at onset age of younger than 45 years; using diagnosis by a rheumatologist as the external standard, the ASAS modifications of the original Berlin algorithm had sensitivities of 77.9% to 79.7% and specificities of 78.3% to 80.4%.^60^ Although the overall performance of this algorithm was favorable, approximately 20% of patients with axSpA were misdiagnosed as not having axSpA. For patients with a high suspicion of axSpA, despite a negative diagnosis per the algorithm, additional imaging may be considered. For example, SIJ MRI may be appropriate in some patients who are x-ray and HLA-B27 negative but have 2 to 3 SpA features, and spondylitis may be considered in symptomatic psoriatic patients without sacroiliitis. Symptom onset later in life would be unusual, although some patients with axSpA may present with loss of spinal mobility rather than back pain as the primary symptom, and more research about onset age is required for axSpA subsets such as axPsA.

## Clinical SpA Feature Evaluation: History and Physical Examination

Clinicians should inquire about SpA features (e.g., IBP, peripheral joint inflammation, enthesitis, uveitis, or response to nonsteroidal anti-inflammatory drugs [NSAIDs]) and arrange for referrals to collaborating specialists, when appropriate, for diagnostic confirmation of suspected uveitis, inflammatory bowel disease, and psoriasis.^61^ Family histories of first- and second-degree relatives with axSpA, psoriatic disease, inflammatory bowel disease, and uveitis are also useful in assessing axSpA risk. A physical examination of patients with axSpA may be completely unremarkable, but clinicians should examine patients for SpA features (e.g., tender and swollen joints, enthesitis, dactylitis, cutaneous psoriasis, psoriatic nail changes) and alternative explanations of back pain (e.g., disk disease, scoliosis). Measurements of spinal mobility, such as the Schober test, are highly variable in healthy individuals and may not be particularly helpful in the diagnosis of axSpA.^62^ Physical examinations for sacroiliitis have low sensitivity and specificity.^63^

## Laboratory Tests

Although no laboratory tests are diagnostic of axSpA, testing for HLA-B27 may be useful in diagnostic assessments (Table 1). Human leukocyte antigen B27 positivity occurs in 70% to 90% of White patients with AS but in less than 10% of the general population^64–66^; however, the prevalence of AS in the White population with HLA-B27 positivity is approximately only 5%.^66^ Studies in patients with nr-axSpA have reported the prevalence of HLA-B27 positivity at 74% to 86%, but these estimates should be interpreted cautiously, because HLA-B27 positivity was used as a criterion for identifying these patients.^29^ Hence, HLA-B27 positivity alone is not diagnostic for axSpA, and a lack of a positive HLA-B27 test does not exclude the diagnosis.^67^ The diagnostic utility of markers of systemic inflammation, such as elevated erythrocyte sedimentation rate or C-reactive protein (CRP), is low; less than 50% of patients with AS present with elevated inflammatory markers at initial presentation, and many other conditions also cause elevations in these inflammatory markers.^68^

## Imaging

For radiographic imaging of patients with chronic low back pain and suspected axSpA, a single, anteroposterior view of the pelvis or a Ferguson view is commonly taken to evaluate for sacroiliitis. Oblique views may arguably provide additional useful information but require slightly higher radiation levels. Changes suggestive of sacroiliitis include sclerosis, erosions, changes in joint width, and ankylosis. Interpretation of sacroiliitis on radiographs can be challenging, and agreement among expert readers is lower when changes are limited to sclerosis or small, localized erosions without changes in joint width or ankylosis.

If the pelvis radiograph of the SIJ is unremarkable or equivocal for sacroiliitis and there is ongoing clinical suspicion for axSpA, a pelvic MRI should be considered. In contrast to plain radiographs or computed tomography (CT), MRI can detect active inflammatory lesions, regardless of structural lesions, and is more sensitive for detecting active sacroiliitis.^39,69^ Currently, the recommended MRI studies include axial and semicoronal sections of the SIJs, T1 sequence (for structural evaluation), and a water-sensitive sequence (to detect inflammation), such as short tau inversion recovery (STIR) or T2-weighted sequence with fat suppression.

The ASAS definition of active sacroiliitis was updated in 2016 to state that active inflammatory lesions of the SIJs should appear as osteitis or bone marrow edema on STIR or T2-weighted images with fat suppression and be clearly present in typical locations, such as the subchondral or periarticular bone marrow.^70^ A positive MRI should show 2 or more bone marrow edema lesions on the same slice or 1 lesion in the same quadrant on 2 or more consecutive slices.^70^ Structural changes may be seen on T1 images, including erosions, partial or complete ankylosis of the SIJs, sclerosis, and fat metaplasia of the subchondral bone.^71^

Although the site of pain is associated with the site of MRI inflammation overall, a limitation of SIJ MRI is that the axial site with the most bone marrow edema may not be the site with the most pain^72^; thus, clinical activity may not consistently correlate with MRI activity.^73^ Also, MRI activity may change over time.^74^ Magnetic resonance imaging reading and interpretation by radiologists and/or rheumatologists are inherently subjective and can vary even among experienced evaluators.^75^ No objective reference could be established during the development of the ASAS criteria.^76–78^ The presence of bone marrow edema on MRI is not specific for axSpA, as it has been observed in patients with mechanical back pain and healthy volunteers.^79–81^ Bone marrow edema fulfilling ASAS criteria was observed among recreational runners, elite ice hockey players, and military recruits before and 6 weeks after intensive physical training^82,83^ and in women with postpartum back pain.^84^ In the SPondyloArthritis Caught Early (SPACE) cohort, a study examining patients with unexplained chronic back pain, 11 of 47 healthy volunteers (23.4%) and 43 of 47 patients with axSpA (91.5%) had MRI lesions consistent with sacroiliitis.^84^ Additional research with MRI and other imaging techniques, such as low-dose CT, may lead to improvements in the diagnostic assessment of axSpA. Low-dose CT shows promise for the evaluation of SIJs, because it demonstrates subtle bony changes better than radiographs, and the radiation risk is low (albeit slightly higher than with radiographs). At the time of writing, the role of low-dose CT in clinical practice remains unclear because of limited availability and uncertain cost.

Spinal imaging is not recommended for axSpA diagnosis in most cases, but spine images may identify alternative causes of back pain, provide prognostic information, and help identify subsets of patients with less-typical axial presentations (e.g., axPsA). If a spinal MRI is deemed appropriate, the sequences should include T1 and water-sensitive sequences (STIR or T2 fat suppressed).

## Underdiagnosis and Diagnostic Delay

Early diagnosis of axSpA is important to minimize disease burden. Importantly, disease activity as measured by the Bath Ankylosing Spondylitis Disease Activity Index and patient-reported pain is high in the early stages of the disease, independent of radiographic changes.^85^ Treatment improves symptoms and physical function,^86^ and patients with AS with a shorter disease duration have been shown to have better response rates than patients with a longer disease duration.^87^ Thus, delayed diagnosis and treatment contribute to the substantial burden of disease on patients.^88^

Although the magnitude of underdiagnosis in axSpA is difficult to quantify, the gap between axSpA prevalence estimates from epidemiological screening research^4^ and health care record data suggests that underdiagnosis is common.^89^ In a National Health and Nutrition Examination Survey, adults representative of the general population of the United States were prospectively screened for axSpA with face-to-face evaluations, and the axSpA prevalence was estimated at 0.9% to 1.4%.^4^ In contrast, chart review studies reported an axSpA prevalence ranging from 0.2% to 0.7%.^89^ This difference in prevalence estimates suggests that axSpA is widely underdiagnosed and underaddressed in our current health care systems. Diagnostic delays are also common. Feldtkeller and colleagues^21^ published a seminal report in 2003 describing an average diagnosis delay from symptom onset of 8.5 to 11.4 years in patients with AS, with a longer delay in patients lacking HLA-B27 positivity. More recently, the estimated delay between AS symptom onset and diagnosis in the United States has been reported to be 14 years.^16^

Reasons for underdiagnosis and diagnostic delays are multifactorial. Patients may be slow to seek care because of the insidious, intermittent nature of symptoms or because of logistical considerations that disproportionately affect young adults (e.g., inadequate insurance, limited flexibility with work schedules). Also, providers may not consider axSpA in patients with back pain, because a relatively small percentage of patients with back pain has axSpA.^9,90,91^ Additionally, the absence of remarkable physical findings or unique biomarkers and the frequent lack of extra-articular manifestations make early diagnosis challenging.^92^ Strategies for screening and referring patients with suspected axSpA to a rheumatologist may be helpful, but they are not practical for many clinical settings in the United States and are infrequently implemented. Thus, processes for identifying and prioritizing appropriate patients for rheumatologist evaluations are often inconsistent or inefficient.^93,94^

Misdiagnoses are also common contributors to diagnostic delays and underdiagnosis among patients with axSpA.^17^ Prior to receiving their axSpA diagnoses, patients often consult with various types of health care providers for unrecognized axSpA symptoms,^17^ thus experiencing unnecessary diagnostic workups and interventions that may delay appropriate evaluations and referrals.^17,95^ Interventions to alleviate pain symptoms may also delay diagnosis.^16^ Importantly, limited accessibility to a rheumatologist leads to diagnostic delays in many communities.^96,97^

## Sex Differences in AxSpA Diagnosis

Women experience longer delays in receiving a diagnosis of axSpA,^18,19,98^ even though studies show that the age at onset does not vary between men and women or that women have a slightly younger age at onset of AS^20,21^; this may reflect erroneous assumptions that women rarely have AS and insufficient awareness of the differences in disease presentations between women and men.^88^ Specifically, enthesitis, patient-reported disease activity, and quality of life were significantly worse in women than in men with AS in several studies.^99–105^ In a study, women reported more pelvic, heel, and widespread pain than men during the course of the disease.^106^ Women may also be more likely than men to receive misdiagnoses of fibromyalgia and psychosomatic disorders.^17^

Women and men also differ with regard to structural changes in the spine and SIJs. Male sex has been associated with more severe radiological progression.^107,108^ Men had more limited chest expansion and increased occiput-to-wall distance than women.^106^ In a 5-year, prospective study of spinal radiographic progression in AS, elevated CRP levels and increased smoking were reported as predictors of structural progression in men but not in women.^109^

Much is to be learned about the reasons that axSpA differs between men and women. Several studies have demonstrated that women and men with AS have different gene expression^110^ and immunologic processes.^111,112^ Despite equivalent interferon γ levels among men and women with AS, higher levels of T_H_17 cells and cellular responses were observed in men.^111^ Additionally, levels of inflammatory molecules, such as vascular endothelial growth factor, interleukin 18, and tumor necrosis factor α, were significantly higher in men with AS than in women in 1 study, which led investigators to believe that men may more frequently experience irregular bone metabolism, leading to osteoproliferation and syndesmophyte formation.^112^ Genetic studies reported a modest to strong association of the *ANKH* haplotype with AS, with more frequent *ANKH* variants on the 3′ end in men and 5′ end in women.^110^ The murine homolog of the ANKH protein, Ank, regulates normal osteogenesis; mutant mice expressing a premature stop codon at the 3′ end of the *Ank* gene develop severe ankylosis.^113^ Understanding sex differences in axSpA is important for decreasing diagnostic delays and misdiagnoses, particularly among women.

## Differential Diagnosis

The differential diagnosis of axSpA is broad; details are discussed below, and a summary is provided in Table 2. Diseases that may be particularly challenging to differentiate from axSpA include causes of chronic back pain beginning in adolescence or early adulthood and diseases that mimic axSpA changes on imaging of the spine or SIJs.

**TABLE 2:** Summary of the Differential Diagnosis of AxSpA

Chronic Back Pain With Onset in Adolescence or Early Adulthood	Distinguishing Features
Degenerative disk disease^114–119^	• MBP exacerbated by flexion ± radicular pain radiating below the knee • Risk factors: obesity, genetics, repetitive microtrauma, other injury • Imaging: disk space narrowing. Disk herniation may be visible on MRI
Fibromyalgia^120–123^	• MBP with widespread pain in other areas of the body, fatigue, and sleep disturbances. Minimal relief with NSAIDs • Risk factors: female sex, obesity, ± adverse childhood experiences • Imaging: no evidence of inflammatory disease or other etiology
Spondylolysis and spondylolisthesis^124–129^	• MBP exacerbated by hyperextension that may extend into buttocks or posterior thighs • Risk factors: activities with repetitive flexion and extension of the lower back (gymnastics, football, ice skating, weightlifting, etc.) • Imaging: with spondylolysis, separation or fracture of the pars interarticularis (pars defect). With bilateral pars defects, the injured vertebra may be shifted forward relative to the vertebra directly below it (spondylolisthesis)
Scheuermann disease^130–132^	• MBP occurring in early adolescence without precipitating trauma • Risk factors: male sex, more severe with tall height • Imaging: vertebral wedging (wider posterior vs. anterior angle) and possible Schmorl nodes (protrusion of disk material into the vertebrae)
Astrocytomas^133,134^	• Gradual or subacute MBP onset, often with subsequent development of sensory or motor dysfunction • Risk factors: genetics, ionizing radiation • Imaging: asymmetrical spinal cord expansion on MRI
**Mimics of AxSpA on Imaging**	**Distinguishing Features**
Osteitis condensans ilii^135–138^	• Asymptomatic or nonradicular low back pain that can extend to the posterior thighs • Risk factors: multiparity, other mechanical stress • Imaging: SIJ sclerosis of the iliac side without erosions or fusion
DISH^139–145^	• Asymptomatic or MBP • Risk factors: male gender, age >50 y, diabetes, obesity • Imaging: flowing bone along the anterolateral vertebral bodies and across the disk space in the thoracic spine with or without lumbar and cervical spine involvement. A lucent area may be seen between the anterior longitudinal ligament and midportion of the vertebral body. SIJs are often unaffected, but the superior aspect of the SIJ may appear fused. Peripheral calcific enthesopathy may occur
Infectious sacroiliitis^146–151^	• Subacute onset of unilateral buttock or back pain with elevated CRP. Fever is usually absent or low grade • Risk factors: IV drug use, pelvic trauma, infectious endocarditis, immunosuppression, cutaneous or genitourinary infection • Imaging: on MRI, unilateral periarticular muscle edema, thick capsulitis, and extracapsular fluid collection may be useful in differentiating infectious sacroiliitis from sacroiliitis due to axSpA
Whipple disease^152–161^	• Large joint migratory arthralgias, abdominal pain, weight loss, and diarrhea, with or without IBP • Risk factors: occupational exposure to soil or animals • Imaging: sacroiliitis and spondylitis indistinguishable from axSpA
Familial Mediterranean fever^143,162–164^	• Intermittent fevers, abdominal pain, large joint arthritis, enthesitis, IBP. Childhood or adolescent onset is typical, but may occur in adulthood • Risk factors: genetics (*MEFV* gene mutations); Turkish, Armenian, North African, Jewish, and Arab descent • Imaging: sacroiliitis indistinguishable from axSpA
Sarcoidosis^149,165–170^	• IBP • Risk factors: sacroiliitis may occur most frequently in sarcoidosis limited to the thorax (thoracic lymph nodes and lungs) • Imaging: sacroiliitis indistinguishable from axSpA
Spinal calcium pyrophosphate deposition disease^171–173^	• Periodic IBP with elevated inflammatory markers • Risk factors: widespread peripheral chondrocalcinosis • Imaging: linear calcium deposition in intervertebral disks, SIJs, and/or peripheral joints
Idiopathic hypoparathyroidism^174–178^	• Hypocalcemia presentation ± back pain • Risk factors: long-standing hypoparathyroidism • Imaging: syndesmophytes. SIJ usually not involved but subchondral bone resorption or sclerosis may occur
Behçet disease^179–188^	• Recurrent mucocutaneous ulcers, ocular inflammation, peripheral arthritis, ± entheseal inflammation • Risk factors: *HLA-B51* gene; Turkish Japanese, Korean, Chinese, Iranian, Iraqi, Saudi Arabia descent • Imaging: sacroiliitis indistinguishable from axSpA
Hereditary hypophosphatemic rickets^189–191^	• Growth limitations, osteoarthritis, back pain, enthesopathies • Risk factors: genetics (autosomal recessive) • Imaging: syndesmophytes without inflammatory spinal lesions beginning in the second or third decade of life. SIJs typically spared
Ochronosis^192–195^	• Hyperpigmentation of skin or sclera, back pain, range-of-motion limitations, and kyphosis beginning in the third decade of life • Risk factors: genetics (autosomal recessive) or exposure to hydroquinone or phenols • Imaging: affects the intervertebral disks and large joints. Ankylosis in the lumbar or thoracic spine may develop later in the disease course. SIJs usually spared

IV, intravenous; MBP, mechanical back pain.

## Causes of Chronic Back Pain With Onset in Adolescence or Early Adulthood

### Degenerative Changes

Spinal degenerative changes are common in young adults. In a study of 20- to 22-year-old patients, 47% had degenerative changes on lumbar MRI, and 52% reported low back pain.^114^ In a population of patients with low back pain younger than 40 years, 38% had degenerative disk changes, including 13% with vertebral end plate spinal changes.^115^ Differentiating degenerative changes from axSpA changes can be challenging.^116,117^ For instance, degenerative changes on end plates may be mistaken for inflammatory lesions, as both are associated with bone marrow edema.^118^ In the Dutch and French early axSpA study populations, the prevalence of spinal degenerative changes on MRI was high and similar in patients with and without axSpA.^118,119^ Despite the high prevalence of degenerative changes and overlapping features of inflammatory and degenerative changes on MRI of the spine, several features can be used to distinguish between degenerative and inflammatory spine disease, including the nature of the back pain, MRI findings (e.g., location of bone marrow edema in the anterior corners of the vertebrae, fatty lesions at the anterior vertebral corners), sacroiliitis, and extra-axial inflammatory arthritis features (e.g., peripheral arthritis, enthesitis, uveitis, HLA-B27 positivity).^118,119^

## Fibromyalgia

Patients with fibromyalgia experience widespread pain, including chronic back pain and tenderness at multiple sites that may mimic enthesitis.^120,121^ However, back pain with fibromyalgia is usually mechanical in nature, rather than inflammatory.^122^ In contrast to patients with axSpA, those with fibromyalgia experience little or no pain relief with NSAIDs.^123^ Furthermore, the sites of maximal point tenderness with fibromyalgia are usually not located precisely at entheseal sites. Differentiation between fibromyalgia and axSpA can be challenging, particularly in patients without definitive imaging findings of axSpA. Fibromyalgia may co-occur with axSpA, with prevalence of fibromyalgia within populations of AS or axSpA ranging from 4% to 25%.^120^

## Spondylolysis and Spondylolisthesis

Among pediatric patients and young adults, spondylolysis manifests as a fracture of the posterior arch in the lower lumbar spine due to overuse (i.e., hyperextension observed in particular athletes), and spondylolisthesis refers to the anterior displacement of a vertebral body due to bilateral defects of the posterior arch.^124,125^ Spondylolysis may progress to spondylolisthesis.^124^ For imaging, MRI is usually preferred due to the lack of radiation exposure. Magnetic resonance imaging detection of a typical spondylolytic lesion requires an edema-sensitive sequence with a STIR or fat-saturated T2 image; a cortex-sensitive image with a T1 or non–fat-saturated T2 sequence; and axial, sagittal, and coronal views.^126^ Magnetic resonance imaging may reveal increased metabolic activity and the presence of a fracture, although further radiographic evaluation may be needed.^127–129^

## Scheuermann Disease

This condition of unknown etiology is characterized by uneven growth of the vertebrae with respect to the sagittal plane, with anterior compression of 5° or greater in 3 or more adjacent vertebral bodies and thoracic spine kyphosis greater than 40° or thoracolumbar spine kyphosis greater than 30°.^130^ Scheuermann disease usually presents in early adolescence and is associated with subacute pain without precipitating trauma.^131^ The pain improves with rest and is worse with activity and extension. Pain often improves with skeletal maturity in adulthood,^131^ but long-term follow-up of adolescents with Scheuermann disease indicates an increased prevalence of back pain in adulthood.^132^ Standing lateral spine radiographs are required for diagnosis, and Schmorl nodes, with protrusion of disk material into the vertebrae, may be observed.^131^

## Primary Tumors of the Spine

Spinal tumors are rare but should be considered in adolescents and young adults with unexplained back pain. Astrocytomas represent the most common spinal cord tumors in both the pediatric and adult populations and typically present with gradual or subacute onset of regional back pain.^133,134^ Astrocytomas can be differentiated from axSpA by the noninflammatory nature of the back pain, sensory or motor dysfunction that may evolve over the course of months or years, and MRI lesions that typically appear as an asymmetrical spinal cord expansion.^133^

## Diseases With Axial Imaging Features That May Mimic axSpA

### Osteitis Condensans Ilii

Osteitis condensans ilii, characterized by benign sclerosis of the ilium adjacent to the SIJ, affects 0.9% to 2.5% of the general population.^135^ It is typically an incidental finding but it can cause nonradicular low back pain,^135,136^ especially in women who have given birth.^135,137^ No fusion or erosion is observed in the SIJ. Subchondral bone marrow edema may be present later in the disease stage.^138^

## Diffuse Idiopathic Skeletal Hyperostosis

Diffuse idiopathic skeletal hyperostosis (DISH) is characterized by flowing hyperostosis at 4 or more contiguous vertebral bodies and the calcification and ossification of soft tissues, principally ligaments and entheses.^139^ Diffuse idiopathic skeletal hyperostosis is mostly seen in men older than 50 years.^140^ In a Hungarian study among those older than 50 years, the prevalence of DISH was 4.9% and 1.4% in men and women, respectively.^141^ Its etiology is unknown but has been linked to metabolic disorders, such as obesity and insulin-dependent diabetes mellitus.^142^ Although most patients are asymptomatic, some experience chronic back pain, stiffness, and limited mobility of the spine.^143^ This condition may affect the SIJ, leading to a suspicion of sacroiliitis, as seen in AS; however, several distinguishing factors may delineate the 2 diseases.^139^ Diffuse idiopathic skeletal hyperostosis tends to involve the superior portion of the SIJ, whereas AS involves the inferior portion. Erosions may be seen in the SIJs of patients with AS, but not in patients with DISH.^139,144^ Diffuse idiopathic skeletal hyperostosis tends to occur on the right side of the spine, and radiolucencies between the calcified anterior longitudinal ligament and the anterior vertebral body may be seen in DISH.^145^ Ankylosing spondylitis and DISH can occur concurrently, although this co-occurrence is infrequently observed.^139^

## Infectious Sacroiliitis

Accounting for up to 4% of bone and joint infections,^146,147^ infectious sacroiliitis is usually seen in children and younger patients.^146^ The time to diagnosis of infectious sacroiliitis may be delayed (average of ≈45 days) because of a lack of specific symptoms; patients usually present with unilateral pain and elevated CRP but do not always have fever.^148^*Staphylococcus aureus* is the primary etiologic agent responsible for this infection; *Streptococcus*, *Escherichia coli*, and *Salmonella* have also been recovered from affected patients.^147,148^ The SIJ is the most commonly reported osteoarticular space for infections with *Brucella*, evidenced by its recovery from synovial fluid.^149^

Musculoskeletal symptoms occurring in infectious sacroiliitis are not easily distinguishable from other causes of sacroiliitis.^149^ Radiographic changes initially show widespread erosions and subsequent bony repair, possibly involving more than the anterior-inferior synovium of the joint; radiographic findings are usually delayed by approximately 2 weeks. Although the findings are not specific, MRI is the imaging technique of choice and will demonstrate intra-articular fluid, bone marrow edema, and periarticular involvement, particularly during the earlier stage of the infection, whereas subchondral sclerosis, erosions, and ankylosis will be apparent in the later stages of the infection.^150,151^

## Whipple Disease

A rare, bacterial infection caused by the gram-positive bacillus *Tropheryma whipplei*, Whipple disease is marked by the lack of inflammatory response to or cytotoxic effects from the infection, suggesting host immune deficiency or immune system downregulation by the bacteria.^152–156^ This condition is commonly characterized by peripheral joint symptoms, which are present in up to 80% of patients.^157,158^ Inflammatory back pain, sacroiliitis, and spondylitis may occur with Whipple disease. Many patients are initially misdiagnosed with enteropathic arthritis or other forms of seronegative inflammatory arthritis, with at least 50% in 2 studies receiving immunomodulatory therapy, including tumor necrosis factor inhibition.^159,160^ A diagnosis of Whipple disease should be considered in patients presenting with the 4 principal manifestations of arthralgias, diarrhea, abdominal pain, and weight loss.^161^

## Familial Mediterranean Fever

Patients with familial Mediterranean fever may have back pain, peripheral arthritis, enthesitis, and imaging changes typical of sacroiliitis.^162–164^ Sacroiliitis more commonly occurs in patients with familial Mediterranean fever with HLA-B27 positivity and/or M694V.^143^ A history of intermittent fevers and serositis may distinguish patients with familial Mediterranean fever from those with axSpA.^143^

## Sarcoidosis

A systemic, chronic granulomatous disease, sarcoidosis commonly affects the skin, lungs, and musculoskeletal system^149^; the prevalence of radiographic sacroiliitis in sarcoidosis ranges from 6% to 14%.^165–167^ Sacroiliac joint involvement in sarcoidosis often occurs with a history of IBP but may present in patients with mechanical back pain.^149,168^ Radiographic evidence of sacroiliitis in sarcoidosis may be similar to that of AS; a biopsy of the SIJ may reveal noncaseating granulomata in the synovium, but SIJ biopsy is usually unnecessary if a sarcoidosis diagnosis can be made via clinical presentation or biopsy of extra-articular tissue.^169^ Sacroiliitis may occur most frequently in sarcoidosis limited to the thorax.^170^

## Spinal Calcium Pyrophosphate Deposition Disease

Although deposition of calcium pyrophosphate dihydrate crystals into fibrocartilage usually occurs in peripheral joints, involvement of the cervical, thoracic, and lumbar spine has been documented.^171^ Patients may experience episodic IBP from acute sacroiliitis with elevated inflammatory markers.^172,173^ Asymptomatic changes to the SIJs that may resemble sacroiliitis from AS may also occur.^171,173^ Linear calcium deposition in the SIJs, intervertebral disks, and peripheral joints is useful in differentiating calcium pyrophosphate deposition disease from axSpA.^171^

## Idiopathic Hypoparathyroidism

Patients with this condition present with various clinical manifestations, including morning stiffness, spinal pain, nail deformities, renal abnormalities, increased bone mineral density, and/or vertical syndesmophyte formation similar to axSpA.^174,175^ Syndesmophytes are associated with long-standing hypoparathyroidism. Sacroiliac joints are usually not involved, but there may be subchondral bone resorption of the SIJ with minimal cartilage irregularities.^174,176,177^ Hypoparathyroidism should be suspected in patients with low serum total calcium or low ionized calcium levels, especially those with a personal or family history of autoimmune diseases.^176,178^

## Behçet Disease

Behçet disease is marked by recurrent mucocutaneous ulcers, ocular inflammation, and peripheral inflammatory arthritis; entheseal inflammation may also occur.^179^ Interestingly, an elevated prevalence of sacroiliitis has been reported in patients with Behçet disease; however, the prevalence has been highly variable, likely due to interobserver variability, and has been more recently shown to be comparable to healthy controls.^180–184^ On imaging, SIJ changes may be indistinguishable in patients with axSpA versus Behçet disease.^181^ Uveitis may occur with both axSpA and Behçet disease, but with axSpA, uveitis is typically limited to the anterior chamber of the eye, whereas uveitis with Behçet disease may also involve the posterior and intermediate ocular chambers.^43,185,186^ Like axSpA, arthritis in medium and large joints is common in Behçet disease, occurring in approximately one-half of patients.^187^ Behçet disease differs from axSpA through a strong association with the *HLA-B51* but not *HLA-B27* gene.^188^

## Hereditary Hypophosphatemic Rickets

X-linked hypophosphatemia, due to a mutation in the phosphate-regulating endopeptidase on the X chromosome (*Phex* gene), is primarily characterized by musculoskeletal anomalies among children and osteoarthritis and enthesopathy among adults.^189,190^ In the late second or third decade of life, adults with hypophosphatemic rickets may experience new bone formation in the spine that resembles axSpA changes.^190^ In contrast to axSpA, patients with X-linked hypophosphatemia do not develop sacroiliitis or inflammatory lesions in the spine, such as erosions or bone marrow edema.^191^

## Ochronosis

Ochronosis is a manifestation of alkaptonuria, a rare, autosomal recessive disorder that causes blue-black pigmentation in skin, sclera, and other connective tissues.^192,193^ Spinal ochronosis, a progressive condition, results from the deposition of the ochronotic pigment within articular cartilages, especially in the dorsolumbar spine, leading to decreased intervertebral disk spaces, disk calcification, and osteopenia; ankylosis may develop spontaneously later in the disease course that may mimic the spinal bony productive lesions of axSpA.^194^ In ochronosis, bamboo spine, annular ossification, joint erosion, and fusion of the SIJs do not occur.^193,195^

## CONCLUSIONS

Over the past decade, the concept of axSpA has broadened to include AS and previously unrecognized axSpA without sacroiliitis on radiographs. This conceptual evolution has spurred research on the early detection and diagnosis of axSpA, revealing differences in disease presentation and severity between men and women, barriers to early diagnosis, and other axSpA-like conditions that may confound diagnosis. The early detection and diagnosis of axSpA, along with breakthroughs in available therapeutic options, have led to improvements in patient care and disease management, but delays in diagnosis and treatment remain common. Additional research and education are critical for recognizing diverse axSpA presentations and optimizing management early in the course of disease.
